# GATA2 deficiency presenting with Hodgkin's lymphoma and cryptogenic organizing pneumonia: a case report of two siblings

**DOI:** 10.3389/fmed.2025.1706109

**Published:** 2025-12-08

**Authors:** Lila H. Abu-Hilal, Yumna Njoum, Ayah Abulehia, Duha I. Barghouthi, Hussam Al-Ameer, Mohammad Bourini, Mohammad Debas

**Affiliations:** 1 Faculty of Medicine, Al-Quds University, Jerusalem, Palestine; 2 Internal Medicine Department, Al-Makassed Hospital, Jerusalem, Palestine; 3 Nephrology Department, Al-Makassed Hospital, Jerusalem, Palestine; 4 Pulmonology Department, Al-Makassed Hospital, Jerusalem, Palestine

**Keywords:** GATA2 deficiency, cryptogenic organizing pneumonia, recurrent infections, genetic mutation, Hodgkin's lymphoma

## Abstract

**Introduction:**

GATA2 deficiency is a rare genetic disorder affecting hematopoiesis, immune function, and the lymphatic system, predisposing individuals to infections and hematologic malignancies. Its link to myeloid neoplasms is well-known, but association with Hodgkin's lymphoma is less explored, and presentation with cryptogenic organizing pneumonia (COP) is extremely rare.

**Case presentation:**

We report two Palestinian siblings with GATA2 deficiency, each presenting distinct complications. The first, a 27-year-old male, had recurrent febrile illnesses, pancytopenia, and progressive lymphadenopathy. He was misdiagnosed with tuberculosis based on imaging and treated for a year before bronchoalveolar lavage and lymph node biopsy confirmed Hodgkin lymphoma. Despite plans for hematopoietic stem cell transplantation, he died from lymphoma complications before the procedure. His 33-year-old brother developed recurrent skin infections, hidradenitis suppurativa, and progressive respiratory failure. Genetic testing confirmed GATA2 deficiency. High-resolution CT showed cryptogenic organizing pneumonia, an uncommon pulmonary manifestation. Following corticosteroid therapy, his condition improved, achieving full resolution over six months.

**Conclusion:**

These cases highlight the variability of clinical presentations in GATA2 deficiency and underscore the importance of considering rare or atypical manifestations. Our observations suggest that Hodgkin lymphoma and COP may rarely occur in the setting of GATA2 deficiency, but further studies are needed to clarify any potential associations. Misclassification of infectious and hematologic disorders can lead to inappropriate treatment and poor outcomes, as in the first case. These observations support considering early genetic testing in patients with unexplained cytopenias, recurrent infections, or atypical pulmonary disease, particularly with a suggestive family history. Early recognition allows timely intervention, personalized surveillance, and potential curative strategies such as hematopoietic stem cell transplantation. Further research is needed to clarify mechanisms linking GATA2 mutations to lymphoproliferative and pulmonary disorders, improving diagnosis and therapy.

## Introduction

GATA2 deficiency is a rare genetic disorder caused by heterozygous mutations in the *GATA2* gene, leading to haploinsufficiency and a broad spectrum of clinical manifestations. These include immunodeficiency, bone marrow failure, myelodysplastic syndrome (MDS), acute myeloid leukemia (AML), and vascular or lymphatic dysfunction. The *GATA2* gene encodes a zinc finger transcription factor critical for hematopoietic stem cell (HSC) maintenance, immune cell differentiation, and lymphatic angiogenesis. The disorder typically presents in late childhood or early adulthood, but its phenotypic variability and overlap with other hematologic and infectious conditions often delay diagnosis (1, 2).

Clinically, GATA2 deficiency is characterized by recurrent and severe infections, particularly with atypical mycobacteria, viruses, and opportunistic fungi. Patients frequently exhibit monocytopenia, natural killer (NK) cell deficiency, and dendritic cell depletion, predisposing them to life-threatening infections and hematological malignancies. While myeloid malignancies such as MDS and AML are well-recognized complications of GATA2 deficiency, Hodgkin lymphoma has rarely been reported. Our case may represent a possible expansion of the known phenotypic spectrum, though a causal link cannot be established based on a single case. Pulmonary involvement is increasingly reported in patients with GATA2 mutations, ranging from opportunistic infections to interstitial lung disease and pulmonary alveolar proteinosis, even in the absence of overt hematopoietic dysfunction. In fact, a significant proportion of GATA2 deficient patients experience lung involvement, with manifestations such as pulmonary alveolar proteinosis (PAP) and pulmonary arterial hypertension (PAH) being observed, and small nodules and reticular infiltrates being common findings on thoracic CT imaging. However, the association between GATA2 deficiency and cryptogenic organizing pneumonia (COP) has not been previously described (3, 4).

Recent studies have expanded the understanding of GATA2 deficiency, highlighting its role in immune dysregulation and its potential association with non-hematopoietic manifestations. For instance, GATA2 mutations have been linked to impaired NK cell function and increased susceptibility to viral infections, particularly human papillomavirus (HPV). Additionally, emerging evidence suggests that GATA2 deficiency may predispose individuals to autoimmune phenomena, further complicating the clinical picture (5, 6).

Herein, we present two Palestinian siblings with adult-onset GATA2 deficiency, one of whom developed Hodgkin lymphoma and the other cryptogenic organizing pneumonia. These cases may represent additional presentations within the broad clinical spectrum of GATA2 deficiency, emphasizing the importance of early genetic testing in patients with unexplained recurrent infections, cytopenias, or atypical pulmonary manifestations.

## Case 1

**Figure 1:**
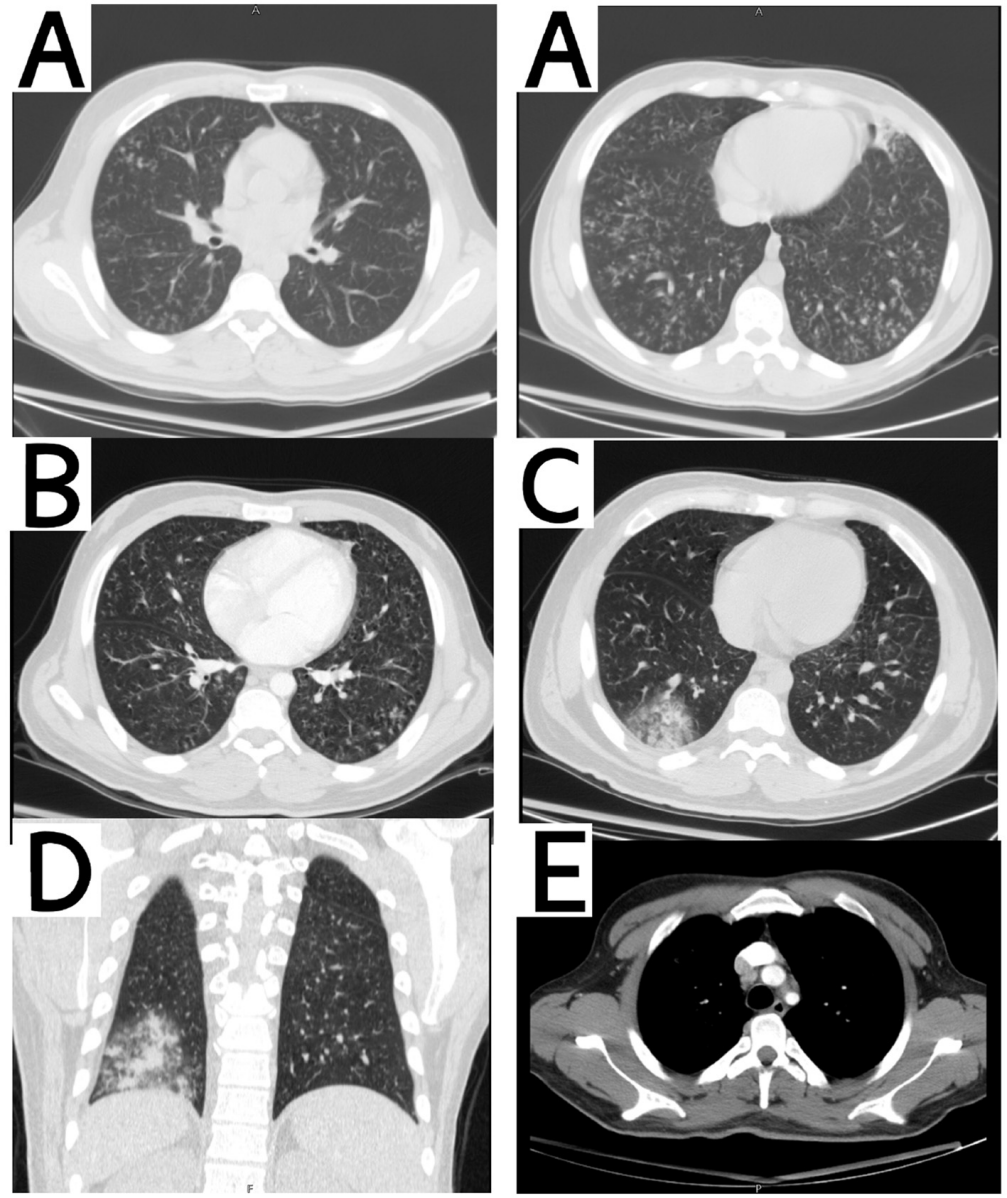
**(A)** Axial chest CT showing miliary nodules and tree-in-bud pattern. **(B)** Axial chest CT following anti-tubercular therapy demonstrating no significant radiological improvement. **(C)** Axial chest CT showing multiple pulmonary opacities, predominantly in the right lower lung zone. **(D)** Coronal chest CT demonstrating multiple opacities, most prominent in the right lower lung zone. **(E)** Mediastinal window revealing enlarged lymph nodes.

A 27-year-old male patient from Palestine presented to the hospital complaining of undocumented fever, night sweats, and chills, which did not improve with treatment for 1 month. Over the course of the following month, he also experienced progressive symptoms including fatigue, epigastric pain, vomiting, dizziness, exercise intolerance, and unintentional weight loss of 10 kg. He had a history of recurrent chest infections with fever and cough, and was treated multiple times with antibiotics and antipyretics. The patient underwent multiple investigations, including complete cell count (CBC), which revealed pancytopenia with severe neutropenia and lymphopenia. The differential count showed WBC 2.44 × 10^9^/L (neutrophils 1.84 × 10^9^/L, lymphocytes 0.49 × 10^9^/L, monocytes 0.05 × 10^9^/L), indicating monocytopenia. Hemoglobin was 9.2 g/dL, MCV 98 fL, and platelets 182 × 10^9^/L. Bronchoscopy revealed focal alveolitis and epithelioid microgranuloma of undetermined significance. He was initially diagnosed with tuberculosis (TB) based on chest computed tomography (CT) scan findings of miliary nodules and tree- in-bud (Figure 1A), and had undergone bone marrow biopsy, which revealed normal cellularity for age and no evidence of granuloma or malignancy. As a result, he was treated as a case of TB for a year due to his recurrent chest infections, night sweating, and weight loss of 35 kg over a year. During follow-up 4 months later, repeat chest CT after anti-tubercular therapy demonstrated no significant radiological improvement (Figure 1B). He was admitted to the hospital, where sputum culture grew *Pseudomonas aeruginosa;* he was treated with intravenous meropenem, resulting in marked clinical improvement.

However, he presented a few months later as his symptoms persisted and intensified, leading to further investigations. Bronchoalveolar lavage (BAL) was done and cultures were negative, ruling out TB. Blood, urine, and sputum cultures revealed the presence of *Pseudomonas aeruginosa*. Due to his persistent fever, chest and abdomen CT were done and showed multiple opacities especially in right lower lung zone, enlarged right supraclavicular lymph node, multiple mediastinal and upper abdominal lymphadenopathies varying in size, with the largest lymph node being 3 × 2 cm with central necrosis, hepatomegaly, splenomegaly and findings suggestive of lymphoma (Figures 1C–E).

Excisional biopsy of the supraclavicular lymph node revealed findings consistent with Hodgkin lymphoma, specifically plasma cell lymphadenopathy with extensive necrosis and scattered large cell CD30 positivity. Additionally, a bone marrow aspiration was performed, revealing a hypocellular specimen, which was likely attributed to marrow failure and pancytopenia. Notably, scattered large cells were observed, potentially indicative of lacunar Hodgkin cells observed in the lymph node biopsy. Following evaluation by an infectious disease specialist, genetic testing was advised, ultimately identifying a heterozygous GATA2 mutation (c.988C>T; p.Arg330). Retrospective review of serology revealed that the patient was EBV-IgG positive and EBV-IgM negative. Unfortunately, the patient's diagnosis occurred late in the disease process, subsequent to the initial lymphoma diagnosis.

Upon referral to a hemato-oncology specialist, the patient was scheduled for an allogeneic hematopoietic stem cell transplant according to international guidelines, following multidisciplinary discussion. The indication for transplantation was based on the pathogenic heterozygous GATA2 nonsense variant, which is predicted to result in premature truncation of the GATA2 protein and is associated with MonoMAC syndrome, myelodysplastic syndrome (MDS), and acute myeloid leukemia (AML) predisposition. Although transplantation is typically indicated for relapsed or refractory Hodgkin lymphoma, in this case, early allogeneic transplantation was recommended as a curative approach for the underlying hematopoietic disorder.

This case underscores the diagnostic challenges posed by atypical clinical presentations of Hodgkin lymphoma, particularly when symptoms overlap with common infectious diseases such as tuberculosis. In this patient, the delay in diagnosis was primarily related to the initial misinterpretation of symptoms and the prolonged focus on an infectious etiology rather than malignancy. Although the underlying GATA2 mutation did not directly obscure classic lymphoma manifestations, its presence remains clinically relevant, as GATA2 deficiency is associated with immune dysregulation and susceptibility to recurrent or refractory infections that may complicate clinical assessment.

This case highlights the need for early reconsideration of alternative diagnoses when patients fail to respond to initial treatment, as well as the importance of multidisciplinary collaboration—particularly between infectious disease specialists and hematologists—to ensure timely evaluation and management. Additionally, early genetic testing should be considered in patients with unexplained infections, persistent cytopenias, or other hematological abnormalities, as identifying underlying germline variants may help guide clinical evaluation and management. Greater awareness and research are needed to improve recognition of rare genetic disorders associated with hematologic malignancies.

## Case 2

A 33-year-old single, unemployed male patient from Palestine, with no significant past medical or surgical history presented to the hospital with a 2-day history of generalized weakness, easy fatigability, dry cough, and pleuritic chest pain mainly at the left side that worsened with deep breaths and was more obvious during cough episodes. He also had chills, rigor, and documented fever of 39 °C that mildly responded to over-the-counter medications and relapsed again after a few hours of taking paracetamol. He had no recent upper respiratory tract infection, contact with ill animals or persons, loss of smell or taste, or recent travel history.

In the last 2 years, the patient has experienced recurrent abscesses with swelling in the axillary area, requiring incision drainage once every 3 months. These abscesses were diagnosed as hidradenitis suppurativa and were treated with topical clindamycin and oral doxycycline. On one visit, he was advised to start on adalimumab (Humira) for his infected hidradenitis suppurativa. He also had persistent skin warts that presented on the dorsal and plantar aspects of his hands and feet for more than 13 years and were not resolved by any traditional or medical intervention.

Upon further investigation, it was discovered that the patient had a brother who died at the age of 27 due to GATA2 deficiency, which was diagnosed based on genetic testing. His brother had recurrent chest infections and was diagnosed with Hodgkin lymphoma. He had two completely healthy sisters, and there was no consanguinity between his parents. Notably, the patient's family was advised to undergo genetic testing regarding GATA2 mutation after their brother's diagnosis, but they didn't follow up due to poor financial status.

Physical examination showed multiple skin lesions on the hands, multiple crops of warts on both the dorsal and plantar aspect of the hands and feet, healed changes in the axillary region from previous hidradenitis suppurativa, and no koilonychia or clubbing. He was a smoker with a history of 15 pack-years and was nonalcoholic.

Based on the patient's family history and recurring symptoms, GATA2 deficiency was suspected and a mutation in the gene was confirmed through further investigation. Single-exome Analysis confirmed the presence of the same pathogenic GATA2 variant (c.988C>T; p.Arg330) previously identified in his brother.

Laboratory investigations showed normocytic anemia (hemoglobin 6.6 g/dL), mild thrombocytopenia (129 × 10^9^/L.), and leukopenia (4.3 × 10^9^/L). Bone marrow biopsy was performed to rule out potential hematological malignancies and revealed a hypocellular marrow (cellularity < 50%) with trilineage hematopoiesis, scattered dysplastic myeloid and erythroid precursors insufficient for a diagnosis of myelodysplastic syndrome, and no leukemic infiltration. Immunohistochemistry was negative for CD34 and CD117 overexpression. Cytogenetic testing [including fluorescence *in situ* hybridization (FISH)] was not performed due to local resource limitations. Therefore, chromosomal abnormalities such as monosomy 7 or trisomy 8—known to carry poor prognostic implications and serve as early indicators for hematopoietic stem cell transplantation—could not be evaluated. However, Single-exome Analysis identified a pathogenic heterozygous GATA2 nonsense variant (c.988C>T; p.Arg330^*^), confirming the molecular diagnosis of GATA2 deficiency. The marrow findings were consistent with an early or low-grade myelodysplastic process associated with GATA2 deficiency (GATA2-related MDS), rather than overt MDS by WHO criteria. The patient was therefore diagnosed with GATA2 deficiency without evidence of hematological malignancy.

**Figure 2:**
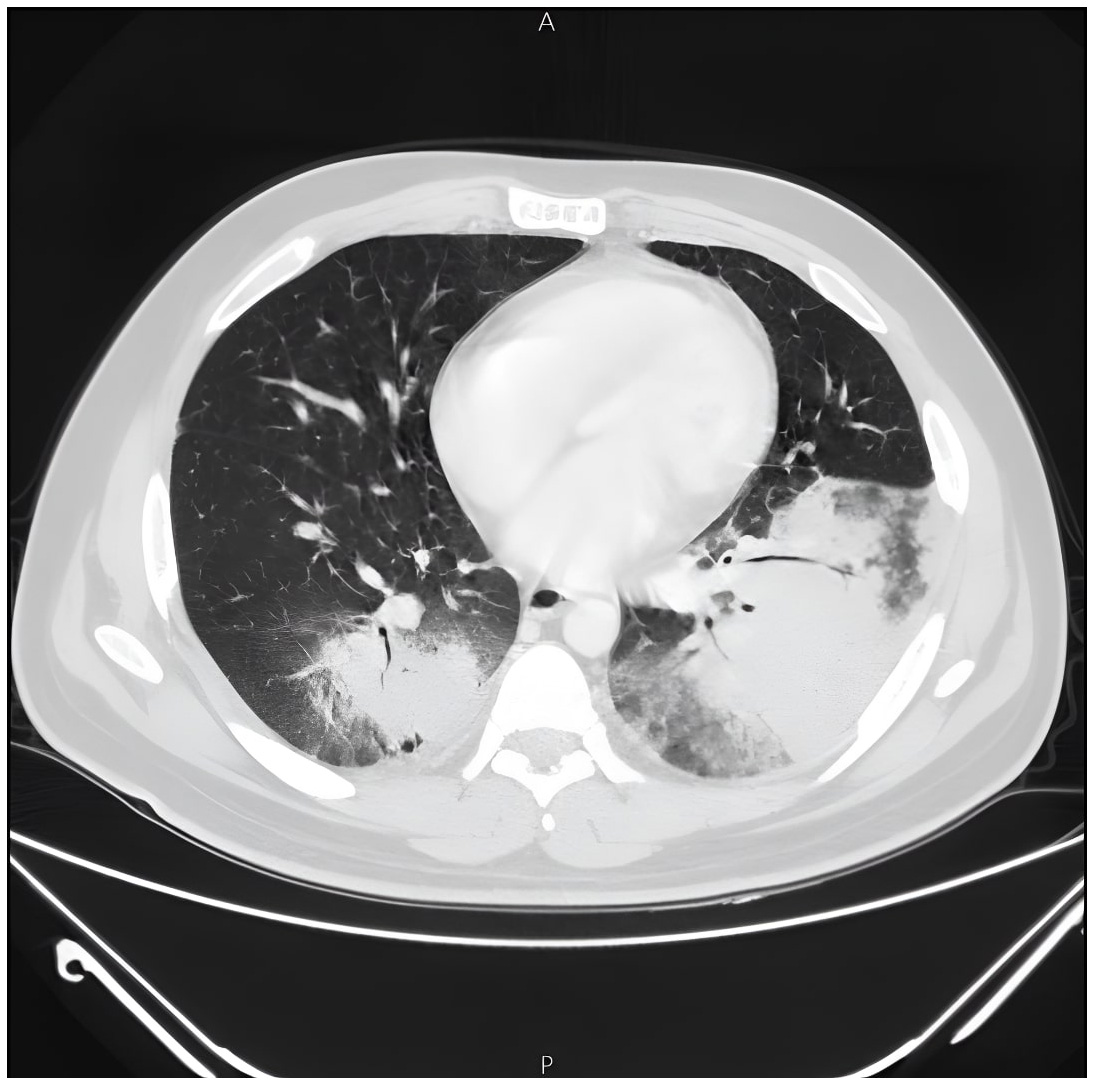
High resolution computed tomography scan of the chest showing patchy consolidations with a predominantly subpleural and/or peribronchial distribution likely suggesting Cryptogenic Organizing Pneumonia.

Clinically, the patient deteriorated, requiring high FiO_2_ support to maintain adequate saturation, and then he became tachypneic even at rest, he also did not respond to antibiotics, including Levofloxacin, vancomycin, sulfamethoxazole-trimethoprim and meropenem. Sputum cultures (performed three times) grew yeast species, while blood and bone marrow cultures were repeatedly negative. High resolution CT scan was performed, which revealed a picture of cryptogenic organizing pneumonia (Figure 2), before he was transferred to the intensive care unit and connected to continuous positive pressure ventilation with close monitoring.

After performing BAL, the samples were sent for culture and sensitivity as well as fluid analysis for cell counting and differential, and TB was ruled out by acid fast staining and negative polymerase chain reaction (PCR) testing. Cytological analysis revealed 60% macrophages and 40% neutrophils, with numerous carbon-laden macrophages. Periodic acid–Schiff (PAS), PASD, Grocott methenamine silver (GMS), and acid-fast stains were negative for microorganisms, and there were no malignant cells. EBV serology was negative.

Consequently, the patient was diagnosed with cryptogenic organizing pneumonia based on the results of the chest computed tomography and excluding other causes. Biopsy could not be taken due to the critical condition of the patient and high risk of the procedure.

However, the unusual presentation of cryptogenic organizing pneumonia was unexpected. He was managed with proper antibiotics, corticosteroids, and supportive care, and he responded well to treatment. He was followed up in the pulmonology clinic. Steroids were weaned over a 6 month period with complete resolution of his cryptogenic organizing pneumonia and he was referred to the hematology clinic for follow up.

This case report describes cryptogenic organizing pneumonia occurring in a patient with GATA2 deficiency, an observation that has not been well reported but cannot be assumed to represent a causal relationship. Clinicians should be aware of the diverse manifestations of genetic disorders and the potential for atypical presentations of pulmonary diseases. Early recognition, accurate diagnosis, and timely intervention are crucial for optimizing patient outcomes in such cases. Further research is needed to explore the underlying mechanisms linking GATA2 deficiency and pulmonary manifestations, providing valuable insights into the management and treatment of these conditions (Table 1).

**Table 1:** Summary of Clinical Characteristics, Investigations, and Outcomes of the Two Siblings with GATA2 Deficiency.

**Feature**	**Case 1**	**Case 2**
Age/sex	27-year-old male	33-year-old male
Presenting symptoms	Fever, night sweats, weight loss (10 kg/month), fatigue, dizziness, vomiting	Fever, pleuritic chest pain, dry cough, generalized weakness, recurrent skin warts, hidradenitis suppurativa
Past medical history	Recurrent chest infections, initially treated as tuberculosis	Recurrent axillary abscesses (hidradenitis suppurativa), chronic warts for 13 years
Initial diagnosis	Tuberculosis (based on CT findings of miliary nodules and tree-in-bud)	Chest infection; later diagnosed as cryptogenic organizing pneumonia
Laboratory findings	Pancytopenia: severe neutropenia and lymphopenia	Anemia (Hb 6.6 g/dL), mild thrombocytopenia, leukopenia
Bone marrow findings	Normocellular for age, no granuloma or malignancy	Hypocellular (< 50%), trilineage hematopoiesis, mild dysplasia, no malignancy
Microbiology	BAL culture: *Pseudomonas aeruginosa*; TB ruled out	BAL: 60% macrophages, 40% neutrophils; all stains negative; EBV negative
Imaging findings	Initial CT: miliary nodules and tree-in-bud pattern; later CT: mediastinal and abdominal lymphadenopathy, hepatosplenomegaly	HRCT: bilateral patchy peribronchial and peripheral opacities suggestive of COP
Final diagnosis	Hodgkin lymphoma secondary to GATA2 deficiency	Cryptogenic organizing pneumonia secondary to GATA2 deficiency
Genetic testing	Genetic analysis revealed a heterozygous pathogenic variant in GATA2 (c.988C>T; p.Arg330).	Same *GATA2* variant (c.988C>T; p.Arg330) confirmed by Single Exome Analysis
Treatment	Planned bone marrow transplant; passed away before procedure due to lymphoma complications	Treated with corticosteroids and antibiotics; complete recovery and stable follow-up
Outcome	Deceased	Alive and clinically stable at follow-up

## Discussion

### Overview of GATA deficiency

GATA2 deficiency is a rare genetic disorder resulting from heterozygous mutations in the GATA2 gene, a crucial transcription factor involved in hematopoiesis, immune function, and lymphatic development (1). These mutations lead to haploinsufficiency, causing a wide range of clinical manifestations.

The GATA2 gene is located on the long arm of chromosome 3 at 21.3 (3q21.3) and consists of 6 or 7 exons depending on the isoform. It encodes a hematopoietic transcription factor with two zinc finger (ZF) domains that bind GATA-DNA motifs in numerous target genes. High GATA2 expression is seen in hematopoietic progenitor cells, early erythroid cells, mast cells, and megakaryocytes. It is also expressed in endothelial cells, the fetal heart and liver, placenta, and central nervous system (6).

GATA2-deficient patients encountered three major mutational types which are missense mutations within ZF2, truncating mutations prior to ZF2, and noncoding variants in the b9.5kb regulatory region of GATA2 (7). Regardless of the several types of mutations leading to GATA2 deficiency, there is no clear association between these mutations and clinical phenotypic expression, except for lymphedema that was linked to the nonsense and deletion mutations (8).

Clinically, GATA2 deficiency has been known to be a major predisposition to myelodysplastic syndrome (MDS). Since its initial description in 2011, phenotypes have ranged from mild cytopenias to severe immunodeficiency and progression to myeloid neoplasia (7, 8).

Dendritic cell, monocyte, B and NK lymphoid deficiency, (MonoMAC) syndrome: monocytopenia and mycobacterial infections, familial myelodysplastic syndrome and acute myeloid leukemia have been described by GATA2 mutations (9). These syndromes are now recognized as different manifestations of a single genetic disorder with protean disease manifestations (7, 9). Syndromic features, such as congenital deafness and lymphedema (indicating Emberger syndrome), or pulmonary disease, are also observed in this patient. The shared clinical feature in all reported cohorts is the propensity for myeloid neoplasia with a total prevalence of approximately 75% and a median age of onset around the second decade of life (7).

Various diseases associated with GATA2 deficiency mutations have been identified, including hematologic diseases, such as MDS (84%), acute myeloid leukemia (14%), chronic myelomonocytic leukemia (8%), pulmonary diseases, such as diffusion (79%) and ventilatory defects (63%), pulmonary alveolar proteinosis (18%), pulmonary arterial hypertension (9%), infectious diseases (70%), disseminated mycobacterial (53%), and invasive fungal infections (16%) (8).

### Pulmonary manifestations

Pulmonary involvement is common in GATA2 deficiency, largely due to recurrent opportunistic infections caused by impaired monocyte/macrophage and dendritic cell function (10). Pulmonary alveolar proteinosis (PAP) occurs in approximately 18% of patients, and pulmonary arterial hypertension in ~9% (8). COP, while observed in our second case, is far less commonly described. Its occurrence may be coincidental or potentially related, although current evidence is insufficient to establish a direct association.

GATA2 plays a role in alveolar macrophage–mediated phagocytosis, and its deficiency may contribute to recurrent infections and reversible pulmonary changes such as PAP and PAH (11). COP is a rare inflammatory and proliferative intra-alveolar process, occurring in about 6.7 cases per 100,000 hospital admissions (12, 13). Diagnosis relies on chest imaging supported by bronchoscopy or biopsy when needed. Although COP is reversible with immunosuppressive therapy, its relationship with GATA2 deficiency requires further study, as available literature does not yet support a consistent association.

## Lymphoid malignancies and immunodeficiency

Patients with diverse primary immunodeficiency diseases (PIDD) were found to have an 8–10 times increased relative risk of lymphoma in comparison with the age-adjusted population. High incidence of malignancies occurred in subjects with PIDD, driven by increased rates of lymphoma in specific PIDD populations, whereas no increased risk of solid tumor malignancies was observed. These findings indicate an important role of the immune system in protecting against specific cancers (14).

In addition to the known association between GATA2 mutation and lymphoma, lymphoma can be caused by immunosuppression- associated overwhelming carcinogenic infections like Epstein-Barr Virus (EBV) Infection.

Up to 25% of people with specific hereditary immunodeficiencies experience a lifelong risk of tumor development, mostly B-cell lymphomas, which can be explained by several mechanisms that can be categorized as intrinsic or extrinsic to the defective immune cell including impaired cellular development, intracellular signaling, chromosomal instability, tissue inflammation, chronic infections, and impaired immune surveillance (15, 16). Although not always easy, differentiating between the different mechanisms is important for employing the most proper management.

In our first case, Hodgkin lymphoma preceded the identification of the underlying GATA2 variant. While lymphoma has been reported in GATA2 deficiency, current data are insufficient to determine whether the relationship is causal or reflects a broader predisposition due to immune dysregulation. The coexistence of Hodgkin lymphoma and GATA2 deficiency in this patient underscores the importance of thoroughly evaluating atypical or severe presentations of lymphoid malignancies.

## Phenotypic variability in familial GATA2 deficiency

Familial cases of GATA2 deficiency demonstrate substantial phenotypic heterogeneity, even among individuals with identical pathogenic variants. In our two siblings, the same variant (c.988C>T; p.Arg330^*^) was associated with Hodgkin lymphoma in one and cryptogenic organizing pneumonia in the other. While this does not establish a causal link between the mutation and either condition, it aligns with prior reports describing striking variability within affected families. For example, Morimoto et al. reported siblings with the same frameshift GATA2 variant presenting with entirely distinct symptoms—Crohn's-like gastrointestinal disease leading to AML in one, and refractory warts with pancytopenia leading to MDS in the other (17). Such reports highlight the unpredictable expression of GATA2 deficiency and the value of early genetic testing and family screening.

## Conclusion

GATA2 deficiency can manifest at any age and with highly variable clinical features. Confirming the diagnosis through genetic testing is essential for appropriate management, preventive care, and family counseling. Management focuses on infection prevention, monitoring for myeloid malignancy, and routine bone marrow surveillance. Hematopoietic stem cell transplantation remains the only curative option, and early referral should be considered in individuals with high-risk features or progressive immunodeficiency.

## Data Availability

The original contributions presented in the study are included in the article/supplementary material, further inquiries can be directed to the corresponding authors.
